# Ag-Coated Heterostructures of ZnO-TiO_2_/Delaminated Montmorillonite as Solar Photocatalysts

**DOI:** 10.3390/ma10080960

**Published:** 2017-08-17

**Authors:** Carolina Belver, Mariana Hinojosa, Jorge Bedia, Montserrat Tobajas, Maria Ariadna Alvarez, Vicente Rodríguez-González, Juan Jose Rodriguez

**Affiliations:** 1 Seccion de Ingenieria Quimica, Facultad de Ciencias, Universidad Autonoma de Madrid, Campus Cantoblanco, E-28049 Madrid, Spain; jorge.bedia@uam.es (J.B.); montserrat.tobajas@uam.es (M.T.); ariadna.alvarez@uam.es (M.A.A.); juanjo.rodriguez@uam.es (J.J.R.); 2 Division de Materiales Avanzados, IPICYT (Instituto Potosino de Investigación Científica y Tecnológica), Camino a la Presa San José 2055, C.P. 78216 San Luis Potosí, Mexico; mariana.hinojosa.reyes@gmail.com (M.H.); vicente.rdz@ipicyt.edu.mx (V.R.-G.)

**Keywords:** ZnO-TiO_2_/delaminated montmorillonite, heterostructures, Ag-coating, solar photocatalytic activity, water purification

## Abstract

Heterostructures based on ZnO-TiO_2_/delaminated montmorillonite coated with Ag have been prepared by sol–gel and photoreduction procedures, varying the Ag and ZnO contents. They have been thoroughly characterized by XRD, WDXRF, UV–Vis, and XPS spectroscopies, and N_2_ adsorption, SEM, and TEM. In all cases, the montmorillonite was effectively delaminated with the formation of TiO_2_ anatase particles anchored on the clay layer’s surface, yielding porous materials with high surface areas. The structural and textural properties of the heterostructures synthesized were unaffected by the ZnO incorporated. The photoreduction led to solids with Ag nanoparticles decorating the surface. These materials were tested as photocatalysts for the degradation of several emerging contaminants with different nitrogen-bearing chemical structures under solar light. The catalysts yielded high rates of disappearance of the starting pollutants and showed quite stable performance upon successive applications.

## 1. Introduction

The development of porous heterostructures based on clay minerals (PCHs) has attracted researchers specially to develop nanoporous materials with predesigned properties for catalytic applications as an alternative to zeolites. In this context, pillared clays (PILCs) were the first studied systems, being prepared by an intercalation of metal-oxopolycations (typically based on aluminum) and subsequently submitted to thermal treatment to consolidate pillars of the metal oxide [1,2]. PILCs have been studied as catalysts because they present high permanent porosity, where the distributed pillars determine, in an ideal perspective, a two-dimensional channel system consisting of micropores comparable to those of zeolites [3]. The possibility of using different types of pillaring agents as well as layered clays of different origins allows for the preparation of PILCs with channels of variable width and pillars of different nature, with potential applications as catalysts in different reactions [4].

Afterwards, new approaches were intended to create other porous clay-derived materials, such as the one reported by Pinnavaia’s group called *Porous Clay Heterostructures* (PCHs) [5]. This method is based on a templated synthesis where a surfactant and a cosurfactant are initially intercalated between the clay layers, changing the hydrophobicity of the clay and creating micelles in the interlayer space. Then, a silicon-alkoxide was incorporated and its further hydrolysis and polymerization was controlled to occur around the micelles. Thus, the silica formed was templated by the micelle, generating silica pillars with a very well-ordered pattern. This methodology has been usually employed to prepare mesoporous silica, with modulate and larger porosity than that achievable by common pillaring strategies [6,7]. The interest of this approach has been extended to the preparation of PCHs involving other atoms than Si, such as Al or Ti, which introduce other functional applications related to catalysis and adsorption [8,9,10].

More recently, a new type of materials related to PILCs and PCHs has been prepared on the basis of oxide nanoparticles (NPs) between delaminated clay sheets, which have been named Delaminated Porous Clay Heterostructures (DPCHs) [11]. This approach uses an organoclay as starting material, which is dispersed in an alcohol, allowing the expansion of the organoclay and the incorporation of alkoxides. Later, the addition of controlled amounts of water provokes the heterocoagulation of the expanded organoclay while the alkoxide is hydrolyzed, giving rise to NPs that remain assembled to the clay network [12,13]. Earlier studies were made on SiO_2_- and SiO_2_-Al_2_O_3_ DPCHs [14], but this strategy has been also applied to prepare TiO_2_-based DPCHs with photocatalytic applications [15,16].

The investigations on TiO_2_-based DPCHs as photocatalysts suggest that the clay can affect the phase of the semiconductor, the size of the NPs formed, and the textural properties of the resulting material. Recent works have demonstrated that the efficiency of these TiO_2_-DPCHs photocatalysts can be improved by doping the TiO_2_ with transition metals or creating TiO_2_-ZnO heterojunctions by a similar synthetic approach that anchors these semiconductors on a delaminated-clay [17,18,19]. These results confirm that the DPCHs’ synthesis can be successfully modulated to obtain photocatalysts active for the degradation of different organic pollutants, with an improved visible absorption capacity and quantum yield. Herein, we report the synthesis of a novel Ag/ZnO-TiO_2_/delaminated clay combining the semiconductor properties of the TiO_2_-ZnO heterojunctions with the light absorption properties of Ag and the porous texture of the delaminated clay. The main aim is to create photocatalysts with enhanced efficiency towards the degradation of emerging contaminants under solar light.

Based on the literature, several clay-based photocatalysts have been reported using layered montmorillonites and TiO_2_ as active phase. Most of them include TiO_2_-PILCs with high surface areas, which have demonstrated high decolorization rates for model dyes under UV light. However, these materials have the TiO_2_ hindering between the clay layers, being an important drawback for their technological application [20]. In fact, the application of TiO_2_-PILCs for the photodegradation of emerging contaminants is not so extended. In this context, different strategies are under study to improve the efficiency of layered clay-based photocatalysts. They are mainly focused on the improvement of the porosity by delaminating the structure, and the anchorage of other semiconductors, such as ZnO, Bi_2_O_3_, silver halides, and other ternary oxides [20], being necessary to study the relation between the semiconductors and clay materials for the photocatalytic reaction.

In this scenario, the DPCHs appear as a promising way to develop photocatalysts with high efficiency. The identification and removal of these pollutants from water receives nowadays special attention. So far, there is no discharge limitation or regulatory status and their effects on human health and the environment are still under study. Among them, pharmaceuticals, pesticides, or personal care products, which of extended world-wide use are appearing in many aquatic environments as well as in wastewater treatment plants, where they are difficult to remove [21,22]. Different technologies are currently under study for these water pollutants, one of them being photocatalysis. It is based on the ability of a semiconductor material to generate electron–hole pairs induced by the absorption of light with an energy greater than its band gap. These charges can be involved in redox reactions allowing the oxidation of many organic molecules [23,24]. This technology appears as a promising way to remove different pollutants because it opens the chance of using solar light as an energy source. The current work focuses its attention on the degradation of pharmaceuticals (acetaminophen and antipyrine) and pesticide (atrazine) as model emerging contaminants because of their frequent use by the population and their impact on the environment [21].

## 2. Experimental

### 2.1. Synthesis of Ag/ZnO-TiO_2_/Clay Materials

The preparation of Ag/ZnO-TiO_2_/clay materials follows the heterocoagulation procedure described for TiO_2_-DPCHs in the literature [16,19]. Summarizing, 1 g of a commercial organo-montmorillonite (Cloisite^®^ 30B supplied by Southern Clay Products, Gonzales, TX, USA) was dispersed in 10 mL of 2-propanol (Panreac, Castellar del Vallès, Spain) under stirring at 50 °C for 24 h. A solution of titanium (IV) isopropoxide (Sigma Aldrich, St. Louis, MO, USA) in 2-propanol (70% *v*/*v*) was slowly added to the clay suspension under stirring, fixing the TiO_2_/clay weight ratio at 2/1. After 15 min, an aqueous solution of zinc acetate dihydrate (Sigma Aldrich, St. Louis, MO, USA) was added dropwise to the slurry, varying the amount of zinc acetate to obtain ZnO-TiO_2_ ratios from 0 to 2% (*w*/*w*). The mixture was kept at 50 °C under stirring until a gel was formed due to the sol–gel transition of the titanium precursor. The gel was first dried at 60 °C for 24 h and the resulting solid was annealed in air at 500 °C for 4 h (with a 5 °C min^−1^ heating rate), thus removing the organic compounds coming from the organo-montmorillonite and the metal precursors. By this way, several ZnO-TiO_2_/delaminated clay solids were prepared.

The incorporation of silver particles was carried out by photoreduction [25]. The appropriate amount of the ZnO-TiO_2_/delaminated clay previously prepared was added to 50 mL of ethanol solution of AgNO_3_, maintaining magnetic stirring for 30 min to ensure the adsorption of the Ag^+^ ions on the solid surface. The suspension was further irradiated at 25 °C with a commercial UV lamp (TecnoLite G15T8, 214 nm, 17 W, Jalisco, Mexico) for 1 h under stirring. Afterwards, the solid was separated by filtration and dried for 18 h at 100 °C. The silver amount was adjusted to 1 and 3 wt % Ag. The final delaminated montmorillonite-coated materials were labeled 1C2T-ZnX-AgY, being X the amount of ZnO incorporated (0.5, 1, 2 wt %) and Y the silver amount deposited (1 and 3 wt %). The solid without ZnO (0 wt % used as reference) was named 1C2T-Ag1 following the same label as that in our previous works [16,17,18].

### 2.2. Characterization of the Solids

The crystal structure of the samples was analyzed with a Bruker D8 diffractometer (Billerica, MA, USA) equipped with a Sol-X energy dispersive detector to obtain the X-ray diffraction (XRD) patterns. Cu Kα radiation in the 2θ range of 2°–70° with a scanning rate of 1.5° min^−1^ was used. The average crystal size (D) was estimated from the (101) diffraction peak of the anatase phase, the most intense peak, using Scherrer’s equation. Wavelength-dispersive X-ray fluorescence spectrometry (WDXRF) was used to determine quantitatively the chemical composition (major and trace elements) of the samples prepared using S8 Tiger Bruker equipment (Billerica, MA, USA). The porous texture of the samples was characterized by N_2_ adsorption-desorption at −196 °C using a Micromeritics TriStar 123 apparatus (Norcross, GA, USA). The samples were previously outgassed under vacuum at 150 °C for at least 8 h. The total surface area (S_BET_) was quantified by the BET method [26], while the external or nonmicroporous surface area (S_EXT_) and the micropore volume (V_MP_) were estimated using the t-plot method [27]. Finally, the total pore volume (V_T_) was calculated from the amount of nitrogen (as liquid) adsorbed at a relative pressure of 0.99.

The band gap values were obtained from the UV–vis diffuse reflectance spectra (DRS) carried out on a Shimadzu UV–vis spectrophotometer (model UV-2600, Tokyo, Japan), with an integrating sphere in the 200–900 nm region, using BaSO_4_ as reference material and the Tauc Plot standard procedure [28]. The representation of (F(R) × hυ)^1/2^ versus hυ (eV) yields a graph with a linear region whose extrapolation to the x axis gives the band gap value. X-Ray photoelectron spectroscopy (XPS) was used to study the surface composition of the catalysts. The XPS spectra were recorded on a K-Alpha-Thermo Scientific spectrometer (Waltham, MA, USA) using Al Kα X-ray (1486.68 eV) as the excitation source. Binding energies corresponding to Ag 3d, Zn 2p, Ti 2p, and O 1s electrons were obtained using as reference the C 1s line, which was taken as 284.6 eV. The fitting of the XPS signals was made by the least-squares method using peaks with Gaussian–Lorentzian shapes. Scanning electron microscopy (SEM, Hitachi S4800, Tokyo, Japan) using secondary electron (SE) and backscattered electron (BSE) detectors was employed for the analysis of the morphology and particle size of the photocatalysts. The transmission electron microscopy (TEM) images were obtained with a TEM 200 kV, Tecnai G220 from FEI COMPANY (Hillsboro, OR, USA) at an accelerating voltage of 200 kV.

### 2.3. Photocatalytic Experiments

The photocatalytic degradation tests were performed using antipyrine (phenazone), acetaminophen (paracetamol), or atrazine (Pestanal^®^) as model compounds. Their respective chemical structures can be seen in Figure S1 of the Electronic Supplementary Information (ESI). The reactions were carried out in 500 mL Pyrex glass reactors (Panreac, Castellar del Vallès, Spain), with two different ports for sampling and flowing air (50 mL min^−1^), under vigorous magnetic stirring. For the tests, these glass reactors were introduced inside a Suntest solar simulator (Suntest XLS+ photoreactor, ATLAS, Mount Prospect, IL, USA) equipped with a 765–250 W m^−2^ Xe lamp which simulates solar radiation. More details of the photocatalytic reaction system are given elsewhere [16]. In a typical experiment, 250 mg L^−1^ of catalyst (Ag/ZnO-TiO_2_/clay) were added to 200 mL aqueous solution containing 5 mg L^−1^ of the corresponding target compound. Before the photocatalytic reaction, the solution was stirred in dark overnight to achieve an adsorption equilibrium. After that, the suspension was exposed to solar irradiation for 6 h. The irradiation intensity was fixed at 450 W m^−2^, and the reaction temperature was monitored to achieve a constant value of 38 ± 1 °C. At given time intervals, 8 mL of the suspension were withdrawn and the photocatalyst was removed by filtration using nylon fiber filters (0.45 μm, Tecknokroma, Sant Cugat del Vallès, Spain). The liquid phase was analyzed by HPLC using a Varian Pro-Start 410 with a UV–vis detector (ProStart 325, Palo Alto, CA, USA) and a reversed phase C18 column (Agilent Technologies, Santa Clara, CA, USA). A mixture of acetonitrile/acetic acid 0.1% *v*/*v* (gradient method: 10/90–40/60% (0–18 min)) was used as the mobile phase, with a constant flow of 0.35 mL min^−1^. The wavelength used for the detection of each compound was 256, 246, and 270 nm (antipyrine, acetaminophen, and atrazine, respectively).

## 3. Results and Discussion

### 3.1. Characterization of ZnO-TiO_2_/Delaminated Montmorillonite Coated with Ag

**Figure 1:**
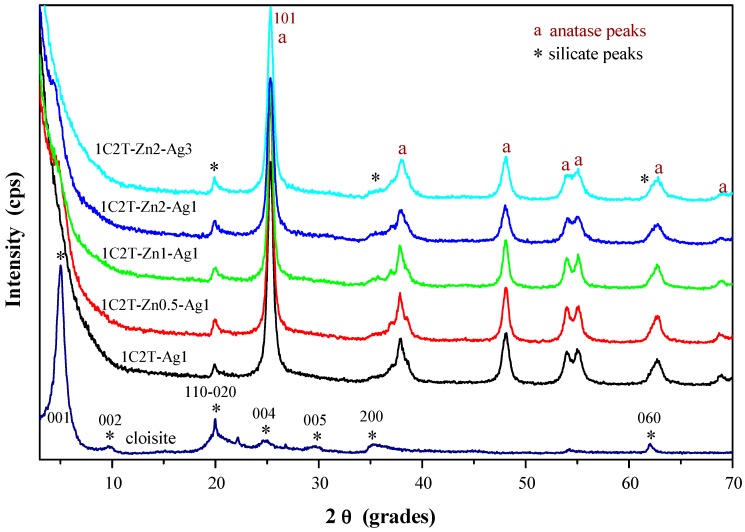
XRD patterns of the starting Cloisite 30B organo-montmorillonite and the synthesized materials.

When the organo-montmorillonite is dispersed in an alcoholic medium it expands, favoring the intercalation of the titanium precursor. Therefore, the subsequent hydrolysis and condensation upon water addition occur between the clay layers, giving rise to the clay delamination. This procedure has been previously reported for several titania and doped-titania solids [16,17,18], being a simple and reproducible way to synthesize delaminated porous clay heterostructures. The delamination suffered by the organo-montmorillonite is seen in the X-ray diffractograms depicted in Figure 1. The raw organo-montmorillonite shows a very intense (001) reflection peak at 1.8 nm (characteristic of this organoclay) that disappears after the incorporation of the titania phase (see 1C2T-Ag1 sample). The lack of the (001) reflection comes with the loss of other (001) reflections, indicating that the montmorillonite sheets are disordered in the c-direction, which can be then associated to its delamination. However, at the same time, it maintained the characteristic layered structure, since the other reflections remained unchanged. This trend was maintained when adding ZnO to the synthesis mixture, so that all of the solids prepared can be described as delaminated clay heterostructures.

It is also noteworthy in Figure 1 that all of the samples depict the characteristic reflection peaks of the anatase phase (JCPDS-78-2486), located at 25.5°, 37.8°, 48.1°, 53.9°, 55.2°, 62.9°, and 68.8° 2θ values. There is not any other peak that can be related to the crystallization of other phases, neither from titanium nor from zinc. In addition, the coating with silver particles did not yield any reflection peak typical of Ag^0^ crystals (38.1° and 44.2° 2θ values), not even in the solid 1C2T-Zn2-Ag3 with the highest amount of silver. This effect can be associated to the low crystallinity of the silver particles, which is below the detection limits of this technique, and to the high monodispersing of the Ag nanoparticles as will be shown later by TEM characterization. The average crystal size of the anatase phase (D) was estimated by Scherer’s equation from the (101) reflection peak. The values are collected in Table 1. With the exception of 1C2T-Zn1-Ag1, the solids yielded similar values (ca. 13 nm), consistent with the anatase size reported previously for the 1C2T heterostructure [16]. This difference can be associated to some small changes in the sol-gel transition caused by the addition of an acid zinc precursor. Usually, the addition of acid during a sol-gel process accelerates the hydrolysis of the alkoxide, changing the subsequent polymerization, which can affect the final crystallization of the titanium oxide [29].

The delamination of the montmorillonite and anatase crystallization yields to the fixation of the TiO_2_ and ZnO, whose percentages are shown in Table 1. The composition of the solids is collected in Table S1 of ESI, where can be seen the SiO_2_, Al_2_O_3_, MgO, and Fe_2_O_3_ contents characteristic of the original cloisite [14]. As expected, when the amount of ZnO increases, the relative amount of TiO_2_ decreases. So, for a better comparison, the ratio between the ZnO and TiO_2_ has been calculated and the results are given in Table 1. These values are close to the theoretical contents estimated during the synthesis process, confirming that the methodology used allows the appropriate control of the amount of ZnO and TiO_2_ incorporated. In this regard, we should point out that although the presence of ZnO cannot be detected by XRD, because of the low amounts incorporated, the chemical analyses demonstrate that the solids have the desired ZnO contents. All solids have been successfully coated with the expected amount of Ag (1 wt %), except the 1C2T-Zn2-Ag3 that shows 2.4% of Ag instead of the 3% estimated. Since the incorporation of Ag particles first involves a saturation of the solid surface with Ag^+^ followed by photoreduction and a final washing step, most probably the surface of the 1C2T-Zn2-Ag3 heterostructure was saturated with the amount fixed and the excess was removed upon the final washing.

**Figure 2:**
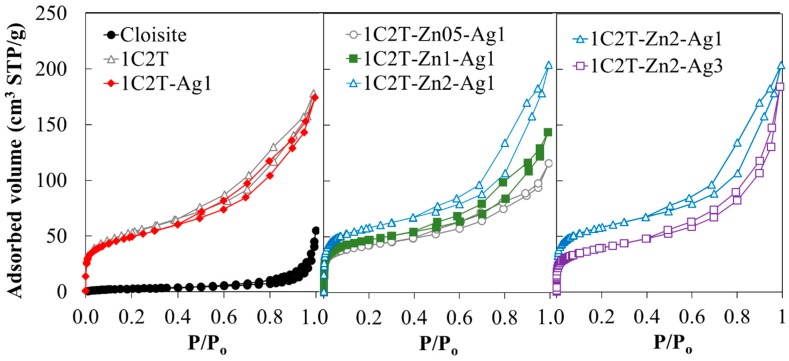
N_2_ adsorption-desorption at −196 °C of the solids.

Figure 2 represents the N_2_ adsorption-desorption isotherms obtained at −196 °C of the different samples, including the Cloisite 30B used as starting organoclay. The isotherms have been separated in order to show more clearly the changes of the porous texture upon the different stages of the synthesis. Cloisite 30B shows a type II isotherm of the UIPAC classification, characteristic of nonporous or macroporous solids [30]. It presents an H3 hysteresis loop associated to the nonrigid aggregates of plate-like particles (e.g., clay materials) [29]. The 1C2T heterostructure has been included as a reference. It shows a fairly different porous texture due to the delamination caused by the introduction of TiO_2_ between the clay layers. It displays a combination of type I and II isotherms also with an H3 hysteresis loop quite common in layered clay-derived materials such as PILCs and DPCHs [3,31]. This kind of isotherm is typical of a widely distributed porous texture with a contribution of micro-, meso-, and macropores due to the “house-of cards” distribution of the plate-like particles [32,33]. The addition of 1% of Ag (see 1C2T-Ag1 isotherm) results in a slight decrease of the amount of N_2_ adsorbed, probably due to a partial pore blockage by Ag particles. Increasing the ZnO content leads to higher amounts of N_2_ adsorbed at a low relative pressure, indicative of a higher micropore volume. This trend can be also related to changes on the sol–gel transition because of the acidity of the zinc precursor. Finally, increasing the Ag incorporated from 1 to 3% decreased the amount of N_2_ adsorbed. As indicated before, this can be associated to a partial pore blockage by the Ag particles coating on the surface of 1C2T-Zn2.

Table 2 summarizes the characterization of the porous texture of the solids. It confirms the significant increase of the specific surface area (S_BET_) of the 1C2T heterostructure with respect to the starting organo-montmorillonite, due to its delamination, which makes accessible both the inner and outer surface of the clay layers [16]. The surface area values are within those previously reported for this kind of DPCH [14,16]. The incorporation of a higher ZnO amount yields a different porous development, as indicated by the increasing values of surface areas and pore volumes (1C2T-Zn0.5-Ag1 < 1C2T-Zn1-Ag1 < 1C2T-Zn2-Ag1). This fact could be associated to the generation of a more disordered porous network, probably related to the different heterocoagulation process occurring upon the sol-gel transition, as indicated before. Regarding Ag incorporation, the values of the surface areas and pore volumes of the corresponding solids confirm the aforementioned partial blockage of porosity by the Ag particles.

**Figure 3:**
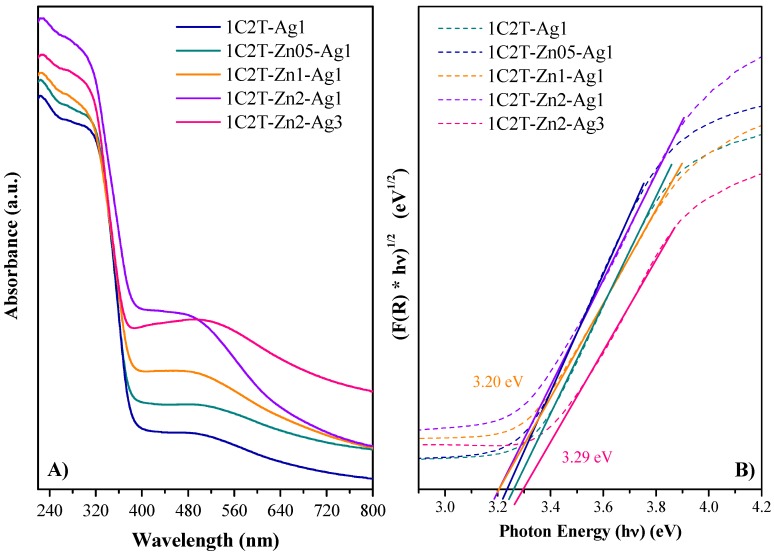
DRS-UV–visible spectra (**A**) and the Tauc Plot (**B**) of the solids.

The DRS-UV–visible spectra of the samples (Figure 3A) show that the characteristic band in the UV region is below 360 nm from the charge transference of TiO_2_, with the absorption edge around 380 nm [34,35]. These profiles also display an absorption shoulder in the visible region, with a maximum at 480 nm, that could be associated to the surface plasmon absorption characteristic of the Ag^0^ particles coating on the solid surface [36]. This absorption shoulder clearly differs according to the ZnO content of the solid, resulting in a sharper absorption in the visible region when the ZnO increases from 0.5 to 2%. This effect suggests a certain type of interaction between the ZnO and the Ag incorporated. Moreover, increasing the silver content from 1 to 3% results in a broader absorption in the visible region, now with the maximum at 510 nm, due to the surface plasmon of the higher silver content. The band gap values (Table 2) were estimated by the Tauc Plot approximation (Figure 3B) considering that these materials are indirect semiconductors as TiO_2_ (their main component) [37]. All of the heterostructures yielded band gap values that were very close together, around 3.2–3.3 eV, without important changes due to the incorporation of the ZnO because both semiconductors, TiO_2_ and ZnO, are characterized by energy band gap values of 3.2 and 3.2–3.4 eV, respectively. Unlike in some other work [38], here the heterojunction made in these solids between TiO_2_ and ZnO did not modify the energy band structure of the final heterostructures.

**Figure 4:**
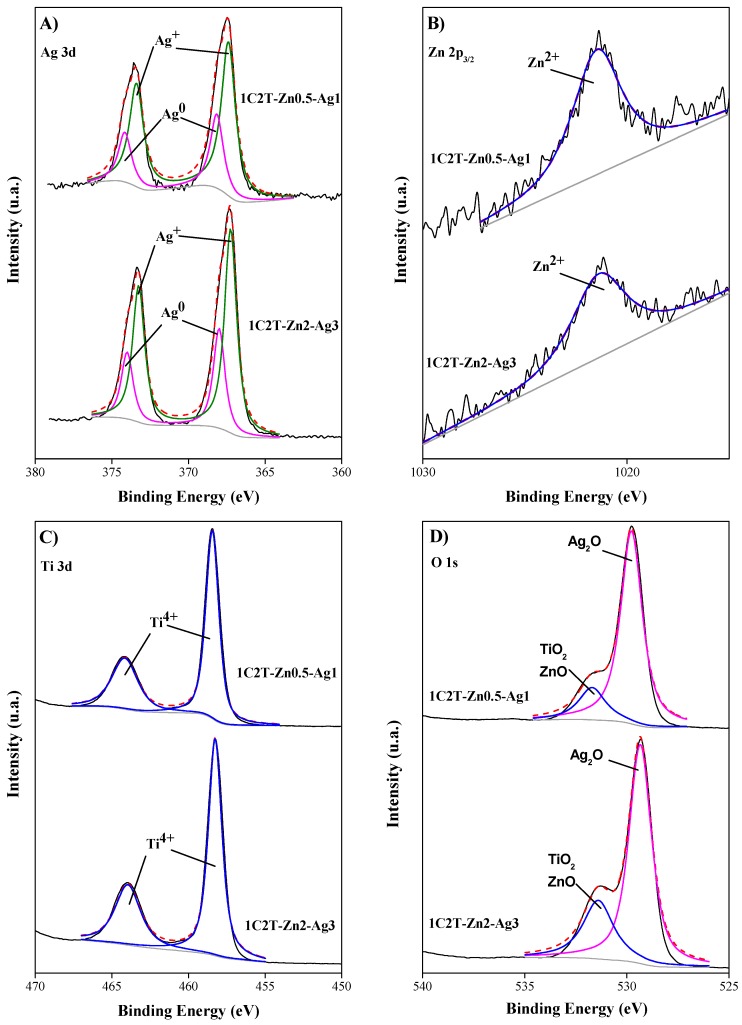
XPS profiles (and their deconvolution) of 1C2T-0.5Zn-1Ag and 1C2T-2Zn-3Ag for: (**A**) Ag; (**B**) Zn; (**C**) Ti; and (**D**) O elements.

The surface composition of the heterostructures was studied by XPS. Figure 4 displays the deconvoluted spectra of the Ag 3d, Zn 2p, Ti 2p, and O 1s regions for the 1C2T-Zn0.5-Ag1 and 1C2T-Zn2-Ag3 solids. The Ag 3d region of the XPS spectra of the samples shows a doublet corresponding to Ag 3d_5/2_ and Ag 3d_3/2_. The Ag 3d_5/2_ peaks located at 368.2 and 368.0 eV for 1C2T-Zn0.5-Ag1 and 1C2T-Zn2-Ag3, respectively, are related to the presence of Ag^0^, while the Ag 3d_5/2_ peaks at 367.4 eV and 367.3 eV for both solids can be attributed to Ag^+^ [39,40,41]. The relative proportions of Ag species of the two solids are given in Table 3, being quite similar for both. The presence of Ag^0^ particles corroborates the visible absorption, being more evident in the 1C2T-Zn2-Ag3 solid because of its higher Ag content. The similar Ag^+^/Ag^0^ ratio is noticeable in spite of the different amounts of Ag (2.4% vs. 1.7% as measured by WDXRF). Although the photoreduction procedure used allows us to coat a solid surface with Ag^0^ [24], the photosensitivity of this specie can result in its oxidation to Ag^+^, which occurs on the surface in a considerably higher proportion [38]. The deconvolution of the Zn 2p_3/2_ profile yielded a peak centered at 1021.4 eV, confirming the presence of Zn^2+^ in both solids [42]. The peak positions of the Ti 2p region, at 464.0 and 458.4 eV, correspond to Ti 2p_1/2_ and Ti 2p_3/2_, in agreement with the presence of Ti^4+^ [43]. The deconvolution of O 1s confirmed the presence of oxygen in different chemical states [44,45]. Two main bands centered at binding energy values around 429.5 and 431.4 eV were observed in all cases, which can be attributed to Ag_2_O and TiO_2_ or ZnO, respectively.

**Figure 5:**
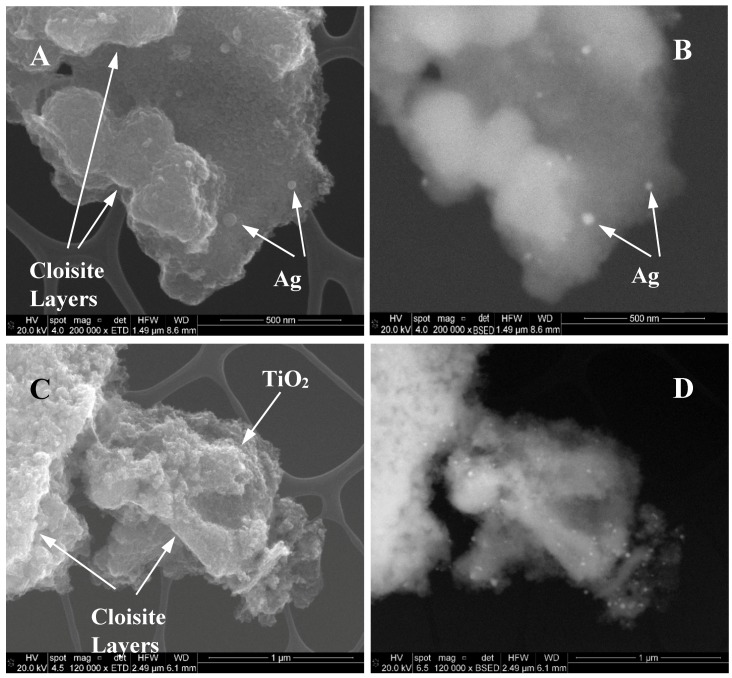
SEM micrographs of 1C2T-Zn0.5-Ag1 (**A**,**B**) and 1C2T-Zn2-Ag3 (**C**,**D**), observed in secondary (**A**,**C**) and back-scattered electrons (**B**,**D**).

Figure 5 shows SEM micrographs of 1C2T-Zn0.5-Ag1 (A and B) and 1C2T-Zn2-Ag3 (C and D) samples observed in secondary (A and C) and back-scattered electrons (B and D). Back-scattered detectors (BSD) provide a much higher mass contrast, therefore the Ag nanoparticles (Ag NPs) appear brighter than the porous support. The images show the presence of the disordered montmorillonite layers, supporting the proper delamination. Furthermore, spongy material can be also observed (Figure 5C) that can be associated to the TiO_2_ phase (with its respective lower amount of ZnO) incorporated between the clay layers. Ag nanoparticles can be observed even in the secondary SEM images (Figure 5A), although they are much more clearly observed in the BSD ones.

**Figure 6:**
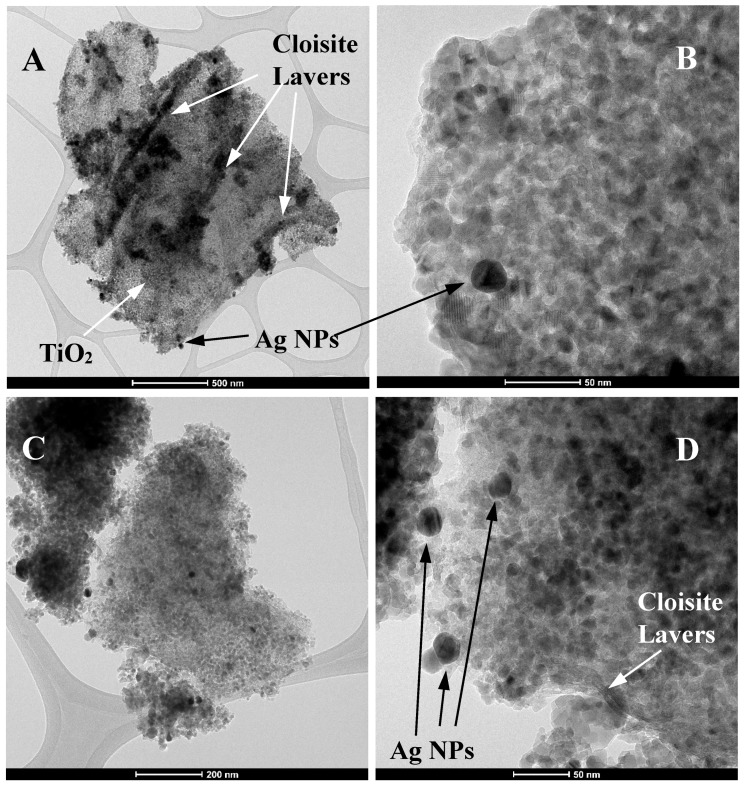
TEM images of 1C2T-Zn0.5-Ag1 (**A**,**B**) and 1C2T-Zn2-Ag3 (**C**,**D**). NPs, nanoparticles.

**Figure 7:**
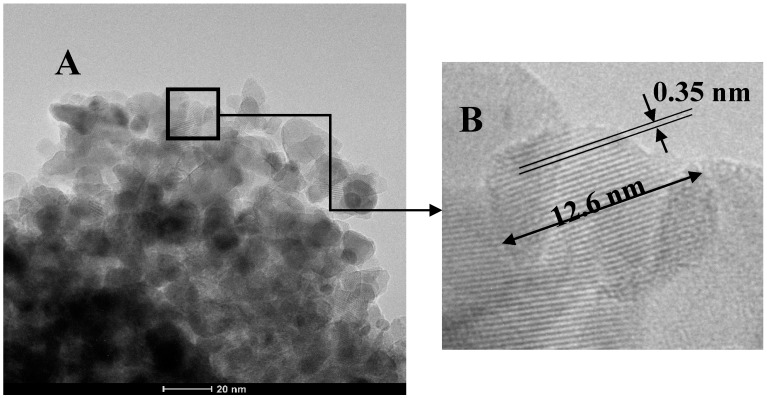
TEM image of 1C2T-Zn2-Ag3 (**A**) and magnification of a TiO_2_ particle (**B**).

**Figure 8:**
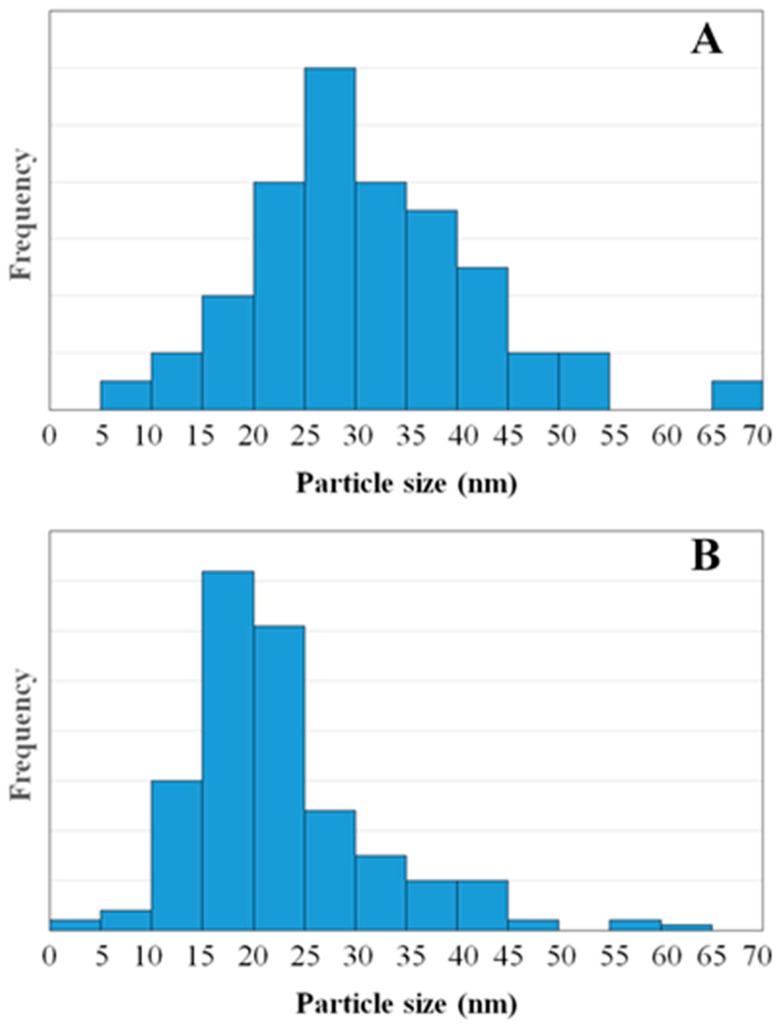
Size distribution of the Ag nanoparticles of 1C2T-Zn0.5-Ag1 (**A**) and 1C2T-Zn2-Ag3 (**B**).

Figure 6 displays some TEM images of 1C2T-Zn0.5-Ag1 (A and B) and 1C2T-Zn2-Ag3 (C and D). Figure 6A shows clearly the presence of different montmorillonite layers surrounded by the TiO_2_ phase. These TEM images show also silver nanoparticles (~20 nm) deposited on the TiO_2_. Figure 7 depicts an additional TEM image of 1C2T-Zn2-Ag3 and a magnification of a TiO_2_ particle (12.6 nm in size), where the lattice space shows a value of approximately 0.35 nm, consistent with that of the anatase phase of TiO_2_ [46]. The TiO_2_ particles in the samples analyzed show sizes between 10 and 15 nm (Figure 7A). Figure 8 represents the size distribution of the Ag nanoparticles of 1C2T-Zn0.5-Ag1 (A) and 1C2T-Zn2-Ag3 (B). Both distributions are monodisperse, being the larger particles close to 70 nm. The mean Ag particle sizes are 31.3 and 21.8 nm for 1C2T-Zn0.5-Ag1 and of 1C2T-Zn2-Ag3, respectively. Some type of interaction between Ag particles and ZnO could favor the higher dispersion of 1C2T-Zn2-Ag3 interaction, as suggested before, from the radiation absorption in the visible region.

### 3.2. Photocatalytic Activity

**Figure 9:**
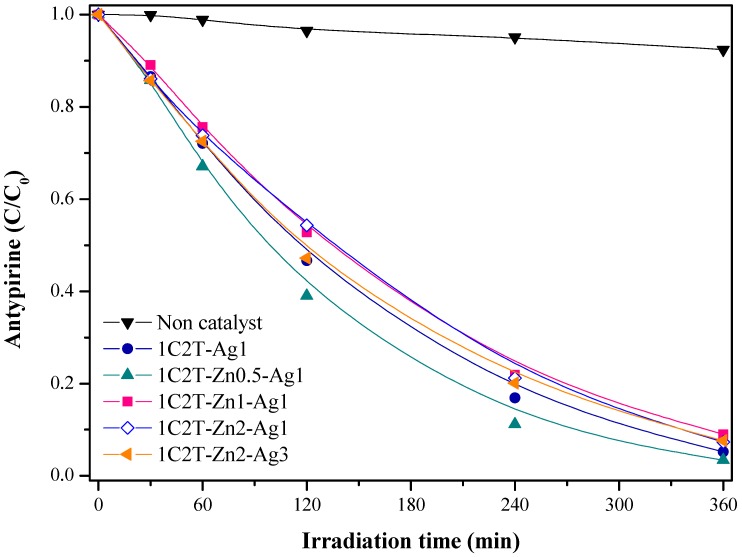
Antipyrine decay upon solar irradiation with the catalysts tested: [Ant]_0_ = 5 mg L^−1^; [cat] = 250 mg L^−1^; W = 450 W m^−2^; T = 38 ± 1 °C.

The photocatalytic activity of the delaminated heterostructures was firstly analyzed for the photodegradation of antipyrine (a model emerging pollutant studied in our previous works [18,19]) under solar light. Prior to the photocatalytic experiments, the adsorption capacity of these materials was checked in dark, showing a low antipyrine adsorption. These results were further used to adjust the initial concentration of the target compound in each photocatalytic test. Experiments in the absence of photocatalyst were also carried out, and it was found that noncatalytic photolysis can be neglected, showing the stability of antipyrine (Figure 9) under solar irradiation.

The evolution of the antipyrine concentration upon irradiation time with all of the Ag/ZnO-TiO_2_ delaminated clay heterostructures is displayed in Figure 9. The photocatalyst 1C2T-Zn0.5-Ag1 degraded nearly 95% of the antipyrine after 360 min, showing higher photocatalytic activity than the 1C2T-Ag1 and the other catalysts with a higher ZnO concentration. The degradation curves were fitted to a pseudo-first-order rate equation, and the resulting values of the kinetic constant (k) are summarized in Table S2 of the ESI. 1C2T-Zn0.5-Ag1 yielded to the highest k value (9.1 × 10^3^ min^−1^), and higher amounts of ZnO did not improve the photocatalytic efficiency, resulting in lower values from 6.5 × 10^3^ to 7 × 10^3^ min^−1^. These values were even higher than those described for similar DPCHs without an Ag coating [19]. Regarding the effect of Ag content, 1C2T-Zn2-Ag1 and 1C2T-Zn2-Ag3 showed similar photocatalytic activity, with almost the same rate constant value (6.9 × 10^3^ min^−1^). Although it was discussed above that the visible absorption of 1C2T-Zn2-Ag3 shifted to higher wavelengths (ca. to 510 nm), that did not cause a positive effect on the photodegradation of antipyrine compared with 1C2T-Zn2-Ag1. Although the characterization of the catalysts indicated that the structural and textural properties of the Ag/ZnO-TiO_2_ delaminated clay heterostructures were similar, the heterojunction between TiO_2_-0.5ZnO and the coating with 1 wt % of Ag gave rise to the best photoefficiency, enhancing the separation of the photogenerated charges and avoiding their recombination. Therefore, this photocatalyst and that with the highest ZnO and Ag contents were used for further studies with the other emerging pollutants.

**Figure 10:**
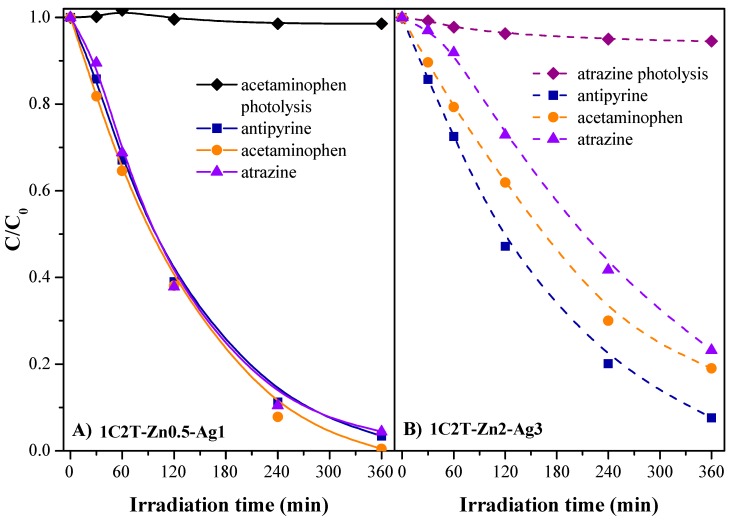
Evolution of the concentration of antipyrine, acetaminophen, and atrazine upon solar irradiation time with 1C2T-Zn0.5-Ag1 and 1C2T-Zn2-Ag3.

A nitrogenated pharmaceutical compound (acetaminophen) and a nitrochlorinated herbicide (atrazine) were also used to test the photocatalytic activity of the materials tested. The chemical structure of antipyrine is derived from pyrazole, a five-membered aromatic heterocyclic containing two nitrogen atoms in contiguous positions, whereas acetaminophen is a p-aminophenol and atrazine contains a chlorinated s-triazine ring. The stability under solar irradiation was firstly checked without photocatalyst (Figure 10), confirming the absence of noncatalytic photolysis. Furthermore, the adsorption capacity of the catalysts was checked for 18 h in dark, showing a low adsorption towards the two contaminants (<5% for acetaminophen and 14% for atrazine). Figure 10 displays the evolution of acetaminophen and atrazine concentrations upon reaction time with the catalysts tested. It can be seen that the highest photocatalytic activity belongs to 1C2T-Zn0.5-Ag1, reaching 92 and 89% degradation of atrazine and acetaminophen after 240 min, and around 96% and complete conversion after 360 min, respectively. Similar photocatalytic activity was found for antipyrine degradation with this catalyst, resulting in similar rate constants for the three contaminants (Table S2 of the ESI). Therefore, the nitrogenated ring structure of the compounds does not cause any noticeable effect on the 1C2T-Zn0.5-Ag1’s photoefficiency under solar light. Nevertheless, the lower activity of the 1C2T-Zn2-Ag3 photocatalyst made clearer the effect of the chemical structure of the contaminants, resulting in the following sequence of degradation rates: antipyrine > acetaminophen > atrazine. This suggests that the pyrazole structure of antipyrine found it easier to interact with the radicals generated during the photocatalytic reaction than did the aminophenol structure of acetaminophen, and much more than the s-triazine ring structure of atrazine. Thus, the influence of the nitrogenated ring structure on the efficiency of the photocatalytic process is noteworthy, but only when a less active photocatalyst is used, since the 1C2T-Zn0.5-Ag1 heterostructure performed efficiently as photocatalyst with the three target compounds tested.

**Figure 11:**
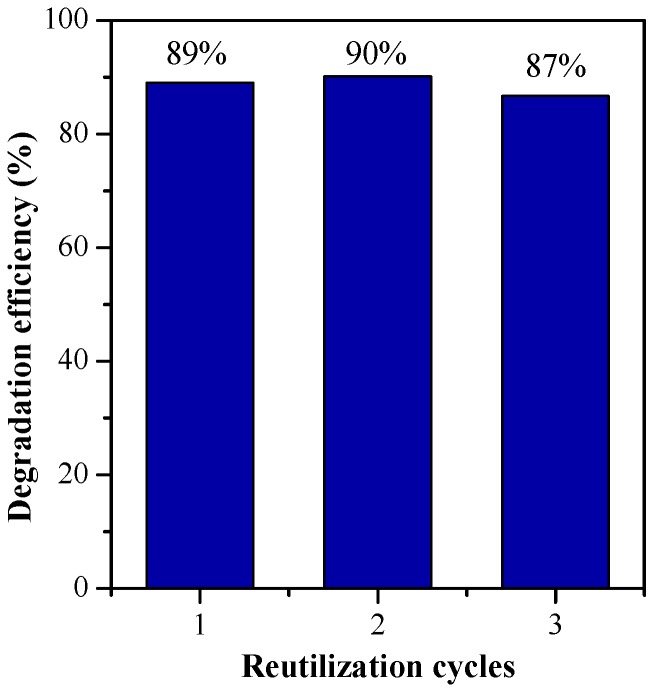
Reutilization test of 1C2T-Zn0.5-Ag1 for the photocatalytic degradation of acetaminophen during three successive runs.

A complementary study was performed to learn about the reusability of the Ag/ZnO-TiO_2_ delaminated clay heterostructures that were synthetized. With this aim, a sequence of three cycles of photocatalytic degradation under solar irradiation during 6 h were carried out using acetaminophen as the target compound and the most active photocatalyst (1C2T-Zn0.5-Ag1). The operating conditions were maintained as in the previously described experiments, and before each run the catalyst was thoroughly washed with water and dried for 1 h at 60 °C. Figure 11 shows the photodegradation percentages reached in the three successive cycles. The photocatalytic activity remained almost unchanged, reaching about 90% degradation. These materials have also the advantage that they can be easily removed from the aqueous solution, showing a fast decantation rate compared with the conventional photocatalysts used as powders.

## 4. Conclusions

Novel heterostructures based on a ZnO-TiO_2_/delaminated montmorillonite coated with Ag nanoparticles, with different ZnO and Ag loads, were successfully synthesized by sol-gel and further photoreduction. The structural characterization revealed, in all cases, the delamination of the montmorillonite and the crystallization of TiO_2_ anatase anchored on the surface of the clay layers. The fixation of ZnO and Ag was confirmed by chemical analyses and XPS, although without the formation of any crystallized phase. The resulting heterostructures exhibited high surface areas with a contribution from both micro- and mesopores. The surface characterization revealed the presence of silver as both Ag^+^ and Ag^0^ at surface concentrations, and the relative amounts were unchanged regardless of the total amount coated. The presence of silver nanoparticles induced the light absorption of the solids in the visible region. These Ag particles displayed an average size of 25–30 nm, showing monodisperse size distributions. All of these heterostructures were effective for the photodegradation of antipyrine under solar light, being 1C2T-Zn0.5-Ag1 the most active. Its efficiency was similar for the three different chemical structures tested as model emerging contaminants (antipyrine, acetaminophen, and atrazine, this last containing also chlorine). The most active catalyst (1C2T-Zn0.5-Ag1) was tested in three successive runs, showing almost unaltered performance.

## Figures and Tables

**Table 1:** Average crystal size of anatase phase (D), chemical composition (wt %), and ZnO/TiO_2_ ratio of the solids synthesized.

Sample	D (nm)	TiO_2_	ZnO	Ag	ZnO/TiO_2_
1C2T-Ag1	12.4	74.30	0.00	1.13	n.d.
1C2T-Zn05-Ag1	13.4	72.70	0.26	0.91	0.36
1C2T-Zn1-Ag1	15.4	71.50	0.49	1.18	0.69
1C2T-Zn2-Ag1	14.0	70.00	1.03	1.17	1.47
1C2T-Zn2-Ag3	13.7	69.80	0.95	2.38	1.36

n.d. not detected.

**Table 2:** Characterization of the porous texture and band gap values of the solids.

Sample	S_BET_ (m^2^ g^−1^)	S_EXT_ (m^2^ g^−1^)	V_MP_ (cm^3^ g^−1^)	V_T_ (cm^3^ g^−1^)	Band Gap (eV)
Cloisite	11	11	n.d.	0.085	n.d.
1C2T	182	152	0.016	0.275	3.21
1C2T-Ag1	173	127	0.021	0.270	3.25
1C2T-Zn0.5-Ag1	144	74	0.033	0.179	3.20
1C2T-Zn1-Ag1	162	83	0.037	0.222	3.20
1C2T-Zn2-Ag1	200	109	0.043	0.316	3.23
1C2T-Zn2-Ag3	138	98	0.018	0.285	3.29

n.d. not detected.

**Table 3:** Surface silver composition (estimated from XPS spectra) of 1C2T-Zn0.5-Ag1 and 1C2T-Zn2-Ag3.

Sample	Ag^+^ (%)	Ag^0^ (%)	Ag^+^/Ag^0^
1C2T-Zn0.5-Ag1	69.5	30.5	2.3
1C2T-Zn2-Ag3	68.5	31.5	2.2

## Supplementary Materials

The following are available online at www.mdpi.com/1996-1944/10/8/960/s1. The supplementary material includes: Table S1: Chemical analyses (wt %) of heterostructures, referred to ignited solids (0% water); Table S2: Values of the rate constant for antipyrine, acetaminophen, and atrazine degradation for the Ag/ZnO-TiO_2_ delaminated clay heterostructures; Figure S1: Chemical structure of the pharmaceuticals and herbicide used as model of emerging contaminants.
