# Supplementary material for: Ag-Coated Heterostructures of ZnO-TiO2/Delaminated Montmorillonite as Solar Photocatalysts
Source: Materials (Basel). 2017 Aug 17;10(8):960. doi: 10.3390/ma10080960 (PMC5578326; doi:10.3390/ma10080960)
Supplement: Supplementary file 1 [file materials-10-00960-s001.pdf]

Electronic Supplementary Information

Ag-coated heterostructures of ZnO-TiO_2/delaminated montmorillonite as solar photocatalysts

C. Belver[a], M. Hinojosa[b], J. Bedia[a], M. Tobajas[a], M.A. Alvarez[a], V. Rodríguez-González[b], J.J. Rodriguez[a]*

[a]Seccion de Ingenieria Quimica, Facultad de Ciencias, Universidad Autonoma de Madrid, Campus Cantoblanco, E-28049 Madrid, Spain

[b]Division de Materiales Avanzados, IPICYT (Instituto Potosino de Investigación Científica y Tecnológica), Camino a la Presa San José 2055, C.P. 78216, San Luis Potosí, Mexico

Table S1. Chemical analyses (wt.%) of heterostructures, referred to ignited solids (0% water).

Sample	TiO_2	ZnO	Ag	SiO_2	Al_2O_3	Fe_2O_3	MgO
1C2T-Ag1	74.30	0.00	1.13	16.40	6.28	1.24	0.61
1C2T-Zn05-Ag1	72.70	0.26	0.91	17.70	6.45	1.33	0.63
1C2T-Zn1-Ag1	71.50	0.49	1.18	17.90	6.55	1.46	0.66
1C2T-Zn2-Ag1	70.00	1.03	1.17	18.50	7.05	1.44	0.67
1C2T-Zn2-Ag3	69.80	0.95	2.38	18.10	6.70	1.32	0.58

Table S2. Values of the rate constant for antipyrine, acetaminophen and atrazine degradation for the Ag/ZnO-TiO_2 delaminated clay heterostructures

	antipyrine	acetaminophen	atrazine
Catalyst	k x 10^3 (min^{-1})	k x 10^3 (min^{-1})	k x 10^3 (min^{-1})
1C2T-Ag1	7.8		
1C2T-Zn05-Ag1	9.1	9.5	9.0
1C2T-Zn1-Ag1	6.4		
1C2T-Zn2-Ag1	6.8		
1C2T-Zn2-Ag3	6.9	4.7	3.8

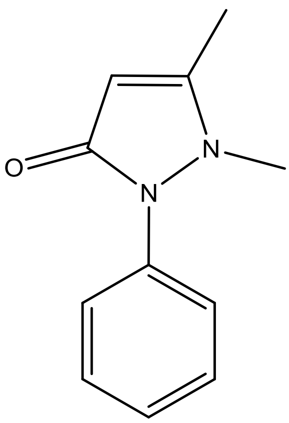

antipyrine

pyrazole derived

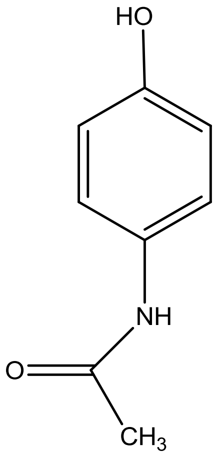

acetaminophen

aminophenol group

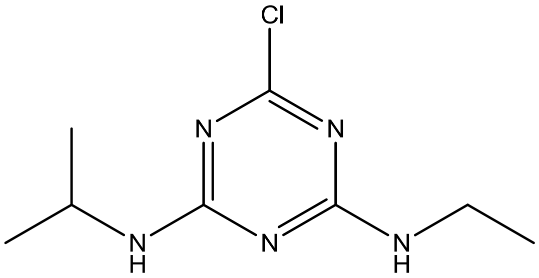

atrazine

nitrochlorinated compound
s-triazine ring

Figure S1. Chemical structure of the pharmaceuticals and herbicide used as model of emerging contaminants.
